# Pathological Physiology, or Clinical, Experimental, and Microscopic Researches upon Inflammation, Tuberculisation, Tumours, the Formation of Callus, &c.

**Published:** 1846-01

**Authors:** 


PHYSIOLOGIE PATHOLOGIQUE, OU RECHERCHES CLINIQUES, EXPERIMENTALES, ET MICROSCOPIQUES, SUR L'INFLAMMATION, LA TUBERCULISATION, LES TUMEURS, LA FORMATION DU CAL, &c. Par *H. Lebert*, Docteur en Medicine et en Chirurgie, &c. Accompagné d'un Atlas de Vingt-deux Planches Gravées. A Paris, 1845.

Pathological Physiology, or Clinical, Experimental, and Microscopic Researches upon Inflammation, Tuberculisation, Tumours, the Formation of Callus, &c. By *H. Lebert*, Doctor in Medicine and Surgery. Two Volumes 8vo. with an Atlas of Plates.

THIS work makes a near approach to what, in former years, would have been called a treatise on morbid anatomy; it differs, however, in this respect, that it comprises "clinical studies, experiments on animals, and microscopic observations," and thus embraces many details of much interest to the pathologist, physiologist, and minute anatomist. The author appears to have a just conception of the mode in which such an inquiry ought to be prosecuted: "It is necessary," he observes, "before approaching the study of pathological anatomy and physiology, to understand the normal structure of the organs and tissues better than it is taught in the schools; it is moreover requisite to learn well the mode of applying the microscope, and to possess exact notions respecting the fundamental forms of organic matter; and, lastly, in order to comprehend the organism when fully formed, it is necessary to follow, step by step, the development of all its constituent parts, to study the inferior organisms of the two sub-kingdoms of organic nature, and to prosecute comparative anatomy, general and microscopic anatomy, and embryology." (*Introduction*, p. x.) It may be proper to premise, that M. Lebert has enjoyed extensive opportunities of carrying on his researches in the Parisian Hospitals, owing to the assistance of MM. Andral, Velpeau, Cruvelhier, Louis, and other distinguished professors.

The first subject treated of relates to the State of the Capillaries, and of the Blood itself in Inflammation. The author, without denying the utility of observations and experiments made on the lower animals, contends that conclusions drawn from this source ought never to be adopted, until it shall have been shown that they are conformable to clinical observation, and to the microscopic examination of the capillaries in inflamed tissues of the human body; he further conceives that these two latter modes of investigation ought to serve as the essential basis of all doctrines relative to the molecular changes produced by inflammation. Agreeing in the necessity of great caution being observed in deducing conclusions from researches confined to animals, we need hardly point out, that this is, after all, the only source from which an actual knowledge of the state of the living vessels in inflammation can be obtained; nor that the important changes induced by death, especially in the state of the blood and its distribution in the several divisions of the vascular system, will always render the evidence of post-mortem examinations a very insufficient test of the condition of the capillaries during life.

After noticing the well-known phenomena occurring on irritation of the small vessels—the accelerated movement of the blood with a temporary narrowing of the capillaries, speedily followed by a retardation of the current, often amounting to a complete stasis, accompanied by a dilated state of the blood-channels, the author points out two remarkable changes, which have, however, been seen and described by other observers. The first of these changes relates to the position of the red particles, which, instead of floating, as they do in health, edgewise, and, speaking of the frog, with their long axis corresponding to that of the blood-vessels, begin to move on their surfaces and with their long diameter turned across the area of the capillaries. The second change noticed is thus described:—"When the stasis is complete, the vessels appear filled with a reddish mass, in which the particles may still be recognised, a circumstance depending on the liquid part of the blood becoming tinged with red; so that, when one of these vessels is opened under the microscope, globules are seen cemented together by a fibro-albuminous liquid, which acquires more and more the tint of hæmatosine. Soon after this, reddish serum transudes through the walls of the capillaries, and thus diffuses the redness all around the vessels in the parenchyma of the inflamed tissue."—(*Tome* I. p. 9.) This coloration of the serum, one of the latest discoveries of minute anatomy, offers a more satisfactory explanation of the most striking among the physical signs of inflammation, redness, than pathology had before possessed. It is doubtless dependent on the laws of endosmose, resembling the passage of the hæmatosine through the walls of the blood-vesicles (a more expressive term of the cell-character than that of particle or corpuscle) when these are placed in water. The phenomenon is not, however, indeed cannot be, the result of simple stagnation, but of some change induced in the qualities of the blood by the inflammatory process; for stoppage of the circulation in the frog's foot, unaccompanied by irritation, does not produce the tinting of the serum.

M. Lebert gives the result of two hundred observations made on the human body, in all their microscopical details, as far as that was possible. He could never seize by direct observation the first effect of inflammation, the acceleration, namely, of the blood and the contracted state of the capillaries; but the other changes—congestion, stagnation, dilatation and tortuosity of the vessels, diffused redness of the inflamed parts owing to the escape of the hæmatosine from the red corpuscles, were, it is affirmed, distinctly recognized. "One of the most ordinary consequences of the turgescence of the small vessels, and of the arrest of the circulation within them, is the rupture of a certain number of these canals. Many reddish excretions, such as the expectoration of pneumonia and the alvine evacuations of dysentery, which were formerly supposed to be merely tinged by the coloring matter of the blood that had simply transuded, show under a microscopic analysis a considerable quantity of the red particles, a certain indication of the rupture of the capillaries."—(*L. c.* p. 13). In the justness of this latter remark we entirely coincide, it having long been our opinion that the blood discs never pass in an entire state through the walls of the minute vessels.

In considering the often-disputed question respecting the formation of new vessels, the author denies that they are ever developed independently of the pre-existing vessels; and, resting on observations made on the tail of the tadpole and on the area vasculosa of the chick, he conceives that in inflammation the obstruction arising from the stagnation in certain vessels, causes an increased pressure upon those in the immediate neighbourhood (and so far he is doubtless correct), the walls of which yielding, form, "by a kind of vagination," lateral prolongations. These vascular arches promptly reaching either the neighbouring capillaries or small veins, readily excavate an opening, being continually pushed on by the same impulsive force of the blood to which they owed their origin. As this subject has been lately fully investigated in this country, especially by Mr. Dalrymple and Mr. Travers, it need not detain us longer than to state that, of all the many theories advanced, the one just noticed seems to us the most mechanical and unsatisfactory.

So much has been of late years written on the microscopic characters of fibrine, the exudation corpuscle, pus, &c., that we shall only briefly notice M. Lebert's observations on the last-named fluid. It is well known that purulent matter consists essentially of a solid portion (pus-globules) and of a serum (liquor puris); it also frequently contains oil-globules, and minute crystals. The serum may be usually obtained by allowing the pus to stand for a few hours in a glass, when the globules subside; or it may be separated by careful filtration. "Thus isolated, the serum is perfectly limpid; it has the colour of white wine, slightly tinged with green; and, although solid matter can no longer be detected in it by the microscope, it retains to a great extent the properties of entire pus. Thus, in injecting it into the vessels of a rabbit, death is caused as if perfect pus were used, only more slowly; dogs in general offer more resistance when pus is thrown into the veins, and the serum alone does not destroy them." (*L. c.* p. 40). The author considers the most important element to be what he terms "*globules pyoïdes*," and which he regards as an imperfectly formed variety of the true pus-globule, but differing from it in their chemical and physical characters: "they are spherical and are composed of two elements; one of which is a somewhat transparent and solid substance, whilst the other consists of molecular granules, from four to ten and upwards in number, irregularly distributed in the interior, which wants, however, the usual characteristic of a nucleus." In examining purulent matter, we have certainly distinguished many globules, in which the interior was filled by numerous granules, the nucleus being apparently absent, and so far we are able to confirm the account given in the text.

The crystals consist of cholesterine and also of other kinds of crystals, usually having the form of elongated prisms, with from three to six lateral faces, and truncated or pointed at the extremities. M. Lebert has also occasionally met with small infusory animalcules, belonging to the genus vibrio of Ehrenberg, in purulent matter derived from various sources, as abscess, carcinomatous ulcer, bronchial expectoration, purulent urine, &c. As the author does not state the exact circumstances under which these minute animals were observed, it is a question whether they were not owing to changes taking place after the pus was extracted, rather than forming an integral part of that fluid.

Before dismissing this subject, it may be desirable to call attention to the relations which exist between pus and mucus, a point which has been principally investigated in connexion with the character of the sputa in various forms of pulmonary disease. Resting on repeated examinations, we hold with those observers, who contend that the distinguishing marks of the pus-globule are in all cases sufficient to indicate that fluid; but, with respect to the microscopic characters of mucus, the evidence is by no means so satisfactory. Up to the present time the existence of some kind of corpuscle in this latter secretion, under the name of the *mucous globule*, is generally admitted, whether this be a modification of the lymph-globule of the blood, of the pus-globule, or a production of the mucous membrane itself. It is thus described by one of the latest writers on microscopy, M. Donné: the mucous globules are small, spherical particles, granular, semi-transparent, slightly fringed in their colour, and $\frac{1}{100}$ of a millimetre in diameter; they appear to be vesicles containing each a nucleus of three or four grains or globulins. In opposition, however, to all these views, and especially to those of the writer just quoted, M. Donné, who has entered minutely into the character of the various mucous secretions, the author contends "that proper mucous globules do not exist, and that normal mucus, free from all accidental mixture, does not contain any of those mucous globules which have been described as very similar to those of pus." M. Lebert thus attempts to explain what he calls the prevailing mistake; in the case of healthy mucus, either young epithelial cells or the large nuclei of the tessellate epithelium have been mistaken for the so-called mucous globules; whilst, as regards morbid secretions, what have been described as globules of mucus, are in fact nothing else than globules of pus.—(*L. c.* p. 68.) It may be well to point out, in reference to this latter position, that the natural secretion of the mucous membranes is readily converted into true pus, merely by an altered action of the part, without ulceration; a fact well stated by Dr. Hodgkin, who remarks, "one of the most remarkable changes which a morbid action produces in the secretion of a mucous membrane, is its acquiring, more or less completely, the characters of true pus; this change takes place independently of any abrasion of the surface of the membrane on which the altered secretion is poured out."

In connexion with the subject of Effusion of Blood in the Brain, M. Lebert states that, in a case where death had ensued eight days after the effusion, examining microscopically the clot situated in the optic thalami and corpora striata, he found the primitive nerve-tubes separated rather than destroyed, and in many places these could be recognised almost intact, a fact "which explains how the functions of the brain might sometimes recover a certain degree of integrity after the absorption of apoplectic extravasations."—(*L. c.* p. 123). We have for a long time felt the necessity of more careful examinations than have yet been made, of sanguineous effusions into the substance of the brain. It frequently happens, in such cases, that the hemiplegia is not perfect; sometimes the patient retains partial power over the fore-arm or leg; at other times, the little and ring-fingers are permanently paralysed, the remaining fingers and the thumb being more or less obedient to the will. Now, in these cases, one of two things, supposing the hemispheres to be, as we believe they unquestionably are, the seat of volition, must have happened; either the effused blood, disrupting all the fibres within its area, must have spared certain of the fibres around, belonging, for instance, to the median nerve; or, a portion of the cerebral tubes contained within the coagulum and going to that nerve, must still have retained their continuity. Our own opinion has been, that the former was the true explanation of the partial voluntary power remaining in these cases, because we have frequently seen a part of the fibres radiating through the corpus striatum and neighbouring parts, pushed, as it were, on one side of the clot, and so avoiding laceration: the facts related by M. Lebert indicate, however, another view of this interesting part of cerebral pathology, and also show the necessity for further researches.

Our limits will not permit us to follow the author in his investigation of the effects of inflammation in the several splanchnic organs; we must, however, make one exception in the case of the *morbus Brightii*, or, as it is here termed, "albuminous nephritis," an affection to which much attention has lately been directed in consequence of a very interesting paper communicated by Dr. George Johnson to the Medical and Chirurgical Society. In that paper, it was contended that the disease essentially depends on a morbid state of the secretory or epithelium cells lining the urinary tubules, consisting of an accumulation of the fatty matter, which, in the form of oil-globules, naturally exists in very small quantities in the above-named cells; thus, in fact, the affection is a fatty degeneration of the kidney, analogous to the fatty degeneration of the liver described by Mr. Bowman, and not an inflammatory disease. It is further affirmed by Dr. Johnson, that this accumulation of fat in the secretory cells necessarily leads to the engorgement of the tubules which they line, and that one or more convoluted tubes thus distended, and projecting on the surface of the gland, or on the surface of a section, constitutes one of the so-called "granulations of Bright." The frequent connexion of albuminous and bloody urine with this disease is attributed to the mechanical compression of the blood-vessels of the Malpighian corpuscles by the dilated tubuli, and not, which is the more common opinion, to the primary vascular turgescence of the organ.* This brief notice of the novel views of Dr. Johnson will enable our readers to appreciate the researches of M. Lebert, who has restricted his inquiries to three questions: firstly, in what state are the elements of the blood found in the urine; secondly, what is the molecular composition of the granular substance; thirdly, in what parts of the kidney are the granulations deposited?" In the commencement of the disease, when the urine has a brownish and slightly reddish tint, many red particles perfectly intact are observed, together with a certain quantity of the colouring matter of the blood, in very irregular fragments; in the more advanced stage, the pure albumen of the blood is detected. The author adds, "these facts prove that the albumen and colouring matter of the blood do not escape from the renal capillaries by simple exosmose, but, as in inflammation elsewhere, the albuminous nephritis offers, at the début,

* We have extracted the above details from a report in the Lancet of Nov. 22, 1845.

and whilst the afflux of blood is most developed, a real rupture of a certain number of capillaries gorged with blood; and, consequently, a portion of the albumen and colouring matter, as well as the particles, escape from the circulating torrent." It does not, however, appear that the author has actually seen the rupture he describes, so that what is stated is rather an inference than an observed fact.

"As to the microscopic molecular composition of the renal granulations of Bright, I have found no other elements than very numerous molecular granules, having a diameter varying from $\frac{1}{1000}$ to $\frac{1}{800}$ of a line, and forming considerable aggregations or globules. It often happens, that a certain number of the above granules are grouped together, and at length are surrounded by a cellular envelope, by a process similar to the formation of the large yolk globules of the hen's egg. I have never been able, after the most cautious observations, to detect these globules in the interior of the capillaries, and I do not believe they are ever formed until their elements have escaped from the capillary vessels by an exosmotic process. Lastly, I have discovered in the globules fatty granules and vesicles, and sometimes in a rather large proportion.

"With respect to the seat of the granular and albuminous extravasations, I have found it to be most frequently in the uriniferous canals, which become, as it were, artificially injected by the effused matter; at other times, the deposit was in the interstitial renal substance, separating the different physiological elements of the kidney from each other."—(1, p. 146.)

The author quotes an observation of Valentin, published in his Repertorium for 1837, in which it is stated, the cortical canals were found to be filled with a greyish-yellow substance. It thus appears that M. Lebert's researches confirm the opinion of those who regard this disease as essentially inflammatory in its type; and, although he has, like Dr. Johnson, seen fatty vesicles within the tubuli uriniferi, he considers them to be merely a secondary product, resulting from the previous congestion of the renal capillaries.

The section on Phlebitis contains many details of interest, inasmuch as they tend to throw some additional light on the cause of those remarkable purulent effusions or metastatic abscesses of the lungs, of the joints, &c., which so frequently result from this obscure affection. The author states that the existing opinions upon this subject may be reduced to three categories:—1. The metastatic purulent collections are merely depôts produced by the pus-globules, which, too voluminous to traverse the finest capillaries, are arrested and become the focus of these abscesses. 2. The blood is the primitive cause both of the phlebitis and of the secondary purulent deposits. 3. The putrid detritus of the tissues detached and absorbed with the pus of the ulcerated or gangrenous parts, becomes the point of departure of the purulent affection. Not feeling satisfied with any of these explanations, M. Lebert has made several experiments on living animals, rabbits and dogs, by injecting pus into the crural artery. The most marked effects were observed in the qualities of the blood; the natural colour was altered to a brownish red tint; in some cases, coagulation was imperfect or absent; in all, the experiments related, nine in number, the red particles and the fibrine "appeared to lose their physiological qualities;" the former became altered in figure, were deprived more or less of their colouring principle, and were diminished in number or entirely disappeared; their place being supplied by very small molecules, and by small elongated bodies: as regards the fibrine, it appeared to be decreased in quantity. It is important to notice that, although the microscope was carefully used with the express purpose of ascertaining if pus could be detected in the blood, the author only succeeded in recognising it in two instances; "once in a manner not to be doubted in the right auricle of one of the animals in which pus had been injected into the femoral artery; and a second time, though doubtfully, in the case of a man who had succumbed under brachial phlebitis."* Another effect of the mixture of pus with the blood in the living animal is the tendency to capillary hæmorrhage; thus, extravasation of blood was almost constantly found in the interlobular substance of the lungs; also in the liver, in the heart, pericardium, kidneys, in the walls of the bladder, &c. These experiments are by no means superfluous, for the cause of the constitutional and other symptoms supervening on phlebitis is not satisfactorily determined. We find, for example, Mr. Hodgson surmising that "it is not improbable that the constitutional irritation may be the effect produced on the nervous system by the pus which is secreted into the vessel being mixed with the circulating blood;" whilst Mr. Travers, arguing against this view, says, "not to insist on the innocuous quality of pus, it should be observed that the most rapidly destructive inflammation is that which has the true adhesive progress, in which no pus is secreted." One of the latest writers on this subject adduces these opinions without deciding the question; for ourselves, we entirely adopt the results of the experimental researches we have just quoted.

M. Lebert seems to have ascertained that a real inflammatory process precedes the local deposit of pus, especially when this occurs in the joints.

"Metastatic abscesses and purulent collections met with in the serous cavities, have this in common, and we insist on the fact, that the parts secreting the pus are always found in a state of inflammation. This may not have been intense; it may, when the disease is prolonged, have disappeared, leaving only its product; but when the formation of these collections and infiltrations of pus is carefully examined, an inflammatory hyperæmia is always detected as a point of departure."

He subsequently adds that, in the parenchymatous organs, he has constantly found, in the vicinity of the principal collection, small deposits, which, being in a nascent condition, offered an interesting subject for analysis. The gradations observed in the progress of these abscesses may be thus briefly enumerated:—1. A local and circumscribed capillary injection, the vessels dilated and gorged with blood more or less coagulated, but without pus-globules. 2. In the centre of this vascularity a yellow point is seen, which is nothing else than a drop of pus, displaying under the microscope some well characterised pus-globules, in general without

---

* This failure in demonstrating microscopically the presence of pus in the blood, is not to be regarded as a proof that purulent matter is not poured into the torrent of the circulation from the inflamed inner surface of the vein. The author himself attributes the effects following phlebitis to such admixture, and several of the English writers who have so well investigated this subject, among whom we may mention Dr. J. Clark and Mr. Wilson, have found pus in the veins implicated.

nuclei, and floating in a serous fluid. 3. A further collection of pus, but still simply infiltrated, so that the tissue of the organ, although apparently destroyed, is still tolerably well preserved; thus, in the liver, the minute biliary canals with their cells, and in the kidney, the uriniferous canals, their epithelium, and the Malpighian corpuscles are recognisable; the vascularity still persists. 4. The purulent infiltration is transformed into a focus, of which the liquid centre, mixed with the debris of the organic tissue, is composed of a granular and fatty pus, there being but few perfect pus-globules. Lastly, by the progress and confluence of several small foci, an abscess, capable of containing a hen's egg or larger, is formed, but differing from an ordinary phlegmonous abscess, by having several lobular anfractuosities.—(P. 288—291.)

These are valuable facts, and although they do not explain the special cause of these remarkable deposits, they disprove the notion of any mechanical transference of pus from the affected vein to the abscess.

The treatment suggested for this fatal disease, which, according to the author, is one of the most frequent causes of death in cases of wounds and operations occurring in hospitals, is the cauterization of the affected vein as advocated, and in some cases successfully applied, by M. Bonnet of Lyons. M. Lebert prefers the actual cautery on account of its immediate action, which is to cause an effusion of lymph, and the plugging up of the vessel, thus assisting the process by which Nature, in some rare cases, effects a spontaneous cure.

Many of our readers are aware that M. Louis appended, in the second edition of his admirable work on Phthisis, an account of the microscopic characters of tubercle, drawn up by M. Lebert. In the present work, this subject is very fully and ably investigated, and will well repay a careful perusal. The author commences with a proposition which will, in some quarters, be received with scepticism: "*it is,*" he affirms, "*a general law in the molecular composition of morbid products, that everything which is really and materially different in pathology, shows this difference in the ultimate elements appreciable by sight, that is in the microscopic structure.*" We have here the expression of a conviction, which must have forced itself on all who have been closely engaged in minute observation; for, although it may be too much to affirm, that the existing knowledge is such as fully to realise our author's assertion, yet the whole tendency of microscopy, normal and abnormal, is to demonstrate as a fundamental law of organization, that wherever there is any, the least, modification of vital action, it is accompanied by a corresponding modification of structure. If it be objected that this principle would require an almost infinite variety of form and construction, we answer by affirming that such variety does in reality exist in animal organisms. He who has seen the multitudinous diversities displayed in the various forms of dentine, in the capillaries of different though closely allied organs, in the several glandular formations, will be prepared to admit not only the possibility, but the great probability of the position above quoted. That in the present day the distinctive microscopic characters of carcinoma, colloid, fungus, are not sufficiently established to serve in all instances as sufficient indications of the disease, may be freely granted; but these and similar imperfections relate to details, not to principles, and the experience of the past fully justifies a confidence in the future, to clear away those conflicting accounts which have shaken the faith of many in the powers of the microscope, as applicable to morbid anatomy, but, we may add, of none who are practically acquainted with the use of the instrument.

The constant elements of tubercle, according to M. Lebert, are, 1, a great quantity of molecular granules, perfectly round, having a diameter varying from $\frac{1}{1600}$ to $\frac{1}{800}$ of a line: 2, a hyaline substance rather consistent, and uniting together the preceding; 3, globules proper to tubercle. The latter constitute the peculiar characteristic of this morbid product, and are thus described: their form is rarely altogether round, although it is probable that, on their first deposition, they approach the spherical figure, and that they assume a less regular, and often angular contour, as we see in so many other analogous instances, from their close juxtaposition; they are of a clear yellow colour, and contain granules, but no distinct nucleus. These tuberculous globules vary considerably in their size, (from $\frac{1}{400}$ to $\frac{1}{250}$ of a line,) but without any definite relation to the age of the subject, or to the organs in which they exist. After contrasting the globules in question with those of pus, cancer, and encephaloid, M. Lebert thus expresses himself: "tubercle then contains, in its crude state, an element which is peculiar to it, and which distinguishes it from all other morbid productions." P. 360.

The remarks of the author on pulmonary tubercle are judicious, and offer many points of interest to the pathologist. He coincides with Laennec and Louis, in opposition to Bayle and other writers, in thinking that the grey granulations of the lungs are not a particular form of phthisis, but that they constitute only one of the forms by which tuberculization begins not only in the lungs, but also in many other organs; and he subsequently adds, that microscopic observation shows that the grey semi-transparent tubercle contains, in fact, all the elements of the yellow tubercles. An important condition of the pulmonic capillaries in this affection is pointed out, and its signification well distinguished.

"They (the grey granulations) are ordinarily surrounded by numerous vascular networks, but it is necessary to avoid taking this vascular injection for a sign of inflammation; it is rather the result of a mechanical hyperæmia, arising from this circumstance, that the grey granulations, usually very numerous, have either caused a great number of the capillary vessels to disappear, or they have displaced those which, prior to their formation, occupied the places in which the tubercular deposits were made. The blood, in consequence, has a smaller containing space for the same quantity, and in this manner the vessels become distended and gorged with that fluid: hence it is certain that this hyperæmia may easily become inflammatory, although this is far from constantly happening." L. c., p. 383.

In a valuable paper on the minute structure of the lungs, published by Mr. Rainey in the last volume of the Medico-Chirurgical Transactions, the cause of the condition of the vessels just noticed is more definitely explained. In pulmonary phthisis the tubercular matter is unquestionably deposited within the air-cells, and, by the gradual increase of the deposit, the vascular plexuses lying between the air-cells are first of all compressed and then absorbed, and thus, as Mr. Rainey states, two pathological states are induced: "first, a portion of lung is rendered impermeable to the

R 2

blood; secondly, the blood is thrown upon the surrounding unaffected parts." A careful examination of well-injected tuberculated lungs confirms this statement, and conveys to the mind of the observer a clear conception of the cause both of the hæmoptysis and of the attacks of inflammation so frequently supervening in phthisis.

In describing the character of the expectoration in tubercular disease, M. Lebert endeavours to prove that the microscopic examination of the sputa does not aid in forming a diagnosis, especially in incipient cases. The author has, however, accurately depicted in the illustrative plates of pulmonary tubercle (Pl. 8, fig. 2, B., and Pl. 9, fig. 1), an appearance consisting of what he terms "fibres pulmonaires," which, we believe, may be received, whenever it can be detected, as a distinctive character of tubercular expectoration. The fibres in question were first accurately described by Mr. Rainey in the paper above-mentioned, as forming the walls of the air-cells where the mucous membrane is absent, and especially as surrounding their apertures in the form of a well-defined border.

The second volume of M. Lebert's work is devoted principally to his researches connected with tumours; it also contains four special memoirs; one on the formation of callus, a second on the vegetable productions met with in tinea, a third on hydatids of the liver, and a fourth on the cellular theory, and on the formation of the elementary parts of the body in health and disease.

With regard to the classification of tumours, the author says, they "may be referred to two categories, of which one comprises those which contain elements that exist normally in the organism; whilst the other includes those composed of elements of a formation altogether new;" among the former, or homœomorphous, are epithelial tumours, encysted, fibrous, erectile, steatomatous, osseous, &c.; the latter, or heteromorphous, includes but one class, namely, cancer.

The application of the microscope as one of the means for arriving at a natural classification of tumours is of modern date; but it promises to become a most important adjunct in this inquiry. Already some interesting results have been obtained, especially in relation to diseased growths of the skin, in confirmation of which, it will suffice to point to the observations made in this country by Mr. Erasmus Wilson, and to those contained in the work before us. Among the valuable observations of M. Lebert, on "Epithelial and Epidermal Tumours," we can only notice those referring to certain morbid growths which are usually regarded as of a malignant and carcinomatous character, but which our author contends are simply epidermoid productions. A tumour of the size of a French-bean firmly adhering to the os tincæ, was excised by M. Lisfranc; on being examined, it was found to be formed of several membranous layers, the outer of which were composed of distinct epithelial cells, (well represented in Pl. X., fig. 1,) whilst the interior consisted of fibres and cellules having the character of epithelial nuclei; vessels were prolonged into the tumour, but were doubtless separated by a limiting membrane, from the cells and nuclei; "it appears, then, that this tumour was primitively due to an epithelial development, and that the vessels subsequently had produced a fibrous structure." Several specimens of staphyloma were in like manner ascertained to depend on hypertrophy of the corneal epithelium.

The author also contends that many tumours of the lower lip reputed cancerous, are in reality pure epidermic tumours ; they are thus described : "the tumours of this kind may acquire a considerable volume, and become tolerably vascular ; they are also susceptible of inflaming, ulcerating, and suppurating. To the naked eye, they are found to be covered, first of all, by a layer more or less thick ; when this is removed, a yellowish liquid, composed of globules of pus and of epidermis, and even occasionally of sebaceous matters, is perceived ; these tumours are in general yellow, or reddish yellow, of an elastic consistence, and composed of papilliform lobules sufficiently elongated and united into groups, presenting a cauliflower aspect. The microscope only shows in these cases the elements of the epidermis and some vessels, and in the crust a mixture of pus and epidermis : the most careful inspection discovers nothing of the elements of cancer, but hypertrophied papillæ are found making a projection into a superficial ulceration and resting on an indurated base, the result of a chronic inflammation." This explanation proving that there is nothing malignant in these tumours, is supposed to account for the operation of excising the lip more frequently succeeding, than in carcinoma of other organs. (*Tome* II., p. 9.)

A similar explanation is suggested in what is called the chimney-sweeper's cancer, originally described by Pott. "I cannot avoid comparing this affection," says M. Lebert, "with the strictly analogous one which we have described as papillary hypertrophy of the lip, and which we have also met with on the skin of the breast and on that of the pubis ; and it is very possible that the chimney-sweeper's cancer is nothing else than a papillary hypertrophy of the skin of the scrotum, having a tendency to ulceration, which, when it is neglected, may extend in surface and in depth, exhausting the powers of the patient and being accompanied by severe suffering." In confirmation of this view, the success of the operation, when performed sufficiently early, is quoted, and likewise the fact that the microscopic examination of one of these affections exhibited epidermic elements.—(*L. c.* p. 327.)

These remarks are full of interest, and support the opinion, that true scirrhus cannot be induced by mechanical or chemical irritation ; in fact, the mode of origin of cancer scroti and of many cases of labial cancer, cannot be reconciled with the pathology of genuine carcinoma.

We regret that our limits will not allow us to notice the researches of the author on the other forms of benign tumours ; we therefore pass on to the heteromorphous or malignant growths, comprising the several forms of cancer. These are distinguished by "having for the essential distinctive element the carcinomatous juice and globules, which differ from all the other forms of globules met with either in the normal state or in other morbid products. The other elements which are also met with in cancer, such as fibres, fusiform bodies, fat, colouring matter, vessels, &c., only constitute accidental elements, and consequently do not present any specific character."—(*L. c.* p. 241.) In a subsequent page, this statement is more strongly urged :—" there are authors of great merit who assert that cancer does not offer peculiar microscopic elements ; we have arrived at a totally different result, and believe we can affirm, that the cancerous globule has tranchant characters which distinguish it from every other kind of morbid products. * * * * * We conceive that these globules ought to be reckoned among those which display the most striking differences from every other kind of cells." The cancerous globule is thus described: it is formed of a membranous envelope or cell, and of a nucleus which encloses a nucleolus; the size is variable, but the mean diameter equals $\frac{1}{100}$ of a line, ranging from about $\frac{1}{130}$ to $\frac{1}{66}$ of a line and upwards. Its form is for the most part round in encephaloid and ovoid, or a little elongated in schirrus; often, however, the globule is far from being regular, and may even present the most varied forms; it is in general more flattened than its nucleus. It is not rare to meet with large concentric cells having thick walls and enclosing several globules disposed in a concentric manner.

Next to the cancerous globules, the element met with in the largest proportion is the fibrous tissue, which may even be the predominant part: the fibres have sufficiently marked contours, are very minute, scarcely exceeding the $\frac{1}{800}$ of a line, and are in general less tortuous than ordinary cellular fibres; in some organs, in the mamma especially, many elastic fibres are seen. The author regards the above fibres, not as a new formation, but as a simple hypertrophy of the cellular tissue of the organ in which the cancer is deposited.

All accurate observers have found crystalline matters in carcinomatous growths, and these, according to M. Lebert, consist of diverse substances, especially of cholesterine; he has also often met with elongated prismatic needles in cancers of the œsophagus, mamma, cervix uteri, and in an encephaloid of the mesentery. In like manner, Müller has detected crystals in considerable number, in some forms of encephaloid and colloid; it is, however, to be remarked that crystalline matter is often found in other tumours and in various excreta.

It is not necessary to follow the author in his account of the other accidental substances recognised in malignant tumours, nor can we notice the very detailed investigations which he has given of the individual forms of cancer. The memoir on the formation of callus contains some details of interest, particularly relating to the respective shares of the periosteum and medullary membrane in the work of reparation (the former being the most active), the microscopic texture of the cartilaginous nidus, the appearance of the Haversian canals, &c.

In the memoir on Tinea is a summary of the various observations that have been made on the development in this disease of some of the simplest forms of cryptogamic plants. Schönlein, guided by the fact ascertained by Audoin, that the silk-worm is infested with a vegetable parasite which is contagious, discovered the intimate nature of the affection, and subsequently the subject has been prosecuted, especially in Germany. There is nothing in the whole range of organization more curious, or more difficult of explanation, than parasitic formations, animal and vegetable. Many of the former more particularly have such habitats, that nothing is known respecting the mode of their development: for example, in addition to the familiar instance of the acephalocysts, there are the filariæ, met with in the subcutaneous areolar tissue (the Guinea-worm) in the bronchial glands, and even in the blood of dogs; the trichina in the substance of muscle; the strongylus in the kidney; the cysticercus in the aqueous humour and beneath the sclerotica of the eye. It is obviously more easy to conceive of the production of those entozoa, which inhabit the cavities of the body having external communications, as the acarus scabei and the entozoa of the healthy sebaceous follicles noticed by Simon of Berlin and since by Mr. Wilson, the various forms of intestinal worms, the spiroptera hominis in the bladder, and even the distoma hepaticum of the gall-bladder; and, in the same way, vegetable parasites may be accounted for, inasmuch as they are, we believe, exclusively found either on the exterior of the body, or in the passages leading from it. A late writer, Sobernheim, has thus enumerated the principal localities of the parasitical cryptogamia in man: "Quevenne and Hannover have found the yeast plant in diabetic urine when it begins to ferment; Hannover, Langenbeck, Bennett, Rayer and Klencke have found a filiform fungus on several mucous membranes. Hannover has seen a species of Leoptomitus Agardh, on the membrane of the mouth and of the tongue in two patients attacked with typhus, and also in the aphthæ of new-born infants, on the mucous membrane of the œsophagus and of the bladder. Bennett has met with a fungus allied to the Penicillum Glaucum in the expectoration of a phthisical patient, and Rayer found vegetations on the pleura and in the intestines of a man attacked with pneumothorax."

In porrigo favosa M. Lebert found "the surface of the favus composed of a membranous envelope of a yellow colour, in which the microscope revealed a homogeneous and finely punctated substance. The interior, of a pale white, was porous and formed of clots entirely composed of sporules and of simple or ramified threads. * * * * They occupy the larger part of the interior of these receptacles, have a round or oval form, with well-defined borders and a homogenous interior. The youngest have a diameter of $\frac{1}{400}$ of a line, whilst those more developed with the same width have a length varying from $\frac{1}{260}$ to $\frac{1}{160}$ of a line." A movement of rotation was observed in these sporules, as they have been indeed in those of Algæ.—(*L. c.* p. 479.) The author after noticing the other form of Porrigo, observes "the vegetable nature of tinea offers a ready explanation of its contagious nature, it is owing to the germination of the sporules in the dermoid tissue of the hair."

We must here conclude our notice of M. Lebert's excellent treatise, which, containing a clear account of the microscopic structure of morbid formations, and being illustrated by a series of accurate and well-executed plates, offers, to all who are interested in the subject, a comprehensive exposition of this new branch of pathology.

